# An Improved Method to Enrich Large Extracellular Vesicles Derived from *Giardia intestinalis* through Differential Centrifugation

**DOI:** 10.3390/life13091799

**Published:** 2023-08-24

**Authors:** Abel Sana, Izadora Volpato Rossi, Bruna Sabatke, Letícia Bassani Bonato, Lia Carolina Soares Medeiros, Marcel Ivan Ramirez

**Affiliations:** 1 EVAHPI—Extracellular Vesicles and Host–Parasite Interactions Research Group, Laboratório de Biologia Celular, Instituto Carlos Chagas-Fiocruz, Curitiba 81310-020, Brazil; sanaabel@ufpr.br (A.S.); izadoravolpato@gmail.com (I.V.R.); nanda.sabatke@gmail.com (B.S.); lcbonato@gmail.com (L.B.B.); 2 Programa de Pós-Graduação em Biologia Celular e Molecular, UFPR, Curitiba 81531-970, Brazil; 3 Programa de Pós-Graduação em Microbiologia, Parasitologia e Patologia, UFPR, Curitiba 81531-970, Brazil; 4 Laboratório de Biologia Celular, Instituto Carlos Chagas-Fiocruz, Curitiba 81350-010, Brazil; liacasm@gmail.com

**Keywords:** *Giardia intestinalis*, extracellular vesicles, differential centrifugation, LEVs enrichment

## Abstract

*Giardia intestinalis* is a flagellated unicellular protozoan that colonizes the small intestine, causing the diarrheal disease called giardiasis. The production of extracellular vesicles (EVs) by *G. intestinalis* and the role of these EVs in the parasite’s interaction with the host have been described. According to biogenesis, EVs are grouped mainly into large (microvesicles—derived from the plasma membrane) and small (exosomes—derived from multivesicular bodies). Populations of EVs are heterogeneous, and improved methods to separate and study them are needed to understand their roles in cell physiology and pathologies. This work aimed to enrich the large extracellular vesicles (LEVs) of *G. intestinalis* in order to better understand the roles of these vesicles in the interaction of the parasite with the host. To achieve the enrichment of the LEVs, we have modified our previously described method and compared it by protein dosage and using Nano tracking analysis. *Giardia intestinalis* vesiculation was induced by incubation in a TYI-S-33 medium without serum, to which 1 mM of CaCl_2_ was added at 37 °C for 1 h. Then, the supernatant was centrifuged at 15,000× *g* for 1 h (15 K 1 h pellet), 15,000× *g* for 4 h (15 K 4 h pellet) and 100,000× *g* for 1.5 h (100 K 1h30 pellet). The pellet (containing EVs) was resuspended in 1× PBS and stored at 4 °C for later analysis. The EVs were quantified based on their protein concentrations using the Pierce BCA assay, and by nanoparticle tracking analysis (NTA), which reports the concentration and size distribution of the particles. The NTA showed that direct ultracentrifugation at 100,000× *g* for 1.5 h and centrifugation at 15,000× *g* for 4 h concentrated more EVs compared to centrifugation at 15,000× *g* for 1 h. Additionally, it revealed that centrifugation at 15,000× *g* 4 h was able to concentrate at the same particle concentration levels as a direct ultracentrifugation at 100,000× *g* for 1.5 h. As for the enrichment of LEVs, the NTA has shown a higher concentration of LEVs in direct ultracentrifugation at 100,000× *g* for 1.5 h, and in centrifugation at 15,000× *g* for 4 h, compared to centrifugation at 15,000× *g* for 1 h. Our results have shown that the most used method at 15,000× *g* for 1 h is not enough to obtain a representative population of large EVs, and we suggest that LEVs released by *G. intestinalis* can be better enriched by direct ultracentrifugation at 100,000× *g* for 1.5 h, or by centrifugation at 15,000× *g* for 4 h.

## 1. Introduction

*Giardia intestinalis* (syn. *Giardia lamblia, Giardia duodenalis*) is a flagellated, anaerobic unicellular protozoan capable of infecting the small intestine of humans [1]. It is divided into eight genetic groups, named A-H assemblages, with sets A and B being the most relevant to human health [2]. The parasite has two main stages during its biological cycle: cyst (infectious, resistant) and vegetative trophozoite [1,2]. A host can become infected by the ingestion of cysts present in contaminated water and food, or by direct fecal–oral contact. The exposure of cysts to the acidic environment of the stomach causes excystation by breaking down the cyst wall, releasing trophozoites into the proximal small intestine. Some trophozoites encyst in the jejunum after exposure to bile fluid. Then, these cysts are shed in the feces and may infect hosts, thereby continuing the cycle [1]. Trophozoites populate the duodenum and replicate extracellularly by binary fission, causing a diarrheal disease known as giardiasis, identified as one of the most frequent diarrheal diseases in the world, with an estimated incidence of 280 million cases annually [2]. Retarded growth of the parasite may, in the long term, result in chronic giardiasis [1]. Most cases are asymptomatic; however, in other cases, the infection can result in diarrhea, malabsorption, abdominal pain, bloating and weight loss. Giardiasis is a multifactorial disease in which various physiological changes caused by trophozoites adhered to the intestinal epithelium of the host can lead to its occurrence [3].

The treatment of giardiasis in humans is based on antiprotozoal drugs from the 5-nitroimidazole family [4]. These drugs cause unpleasant side effects and resistance of the parasite to drugs. Giardiasis treatments with metronidazole, tinidazole and albendazole fail at a rate of approximately 20% [5]. So far, there is no effective vaccine against *G. intestinalis* as the parasite constantly changes its variant surface proteins allowing it to evade the immune system, which makes vaccine development challenging [5].

The lack of an effective treatment and vaccine against *G. intestinalis* has driven the search for new, important targets in the interaction of the parasite with host cells. This search may open the way for the development of new therapies. Among the components involved in the host–pathogen interaction that have been widely explored are the extracellular vesicles (EVs).

In recent years, intercellular communication mediated by EVs has received attention due to its potential to carry biomolecules and trigger signals in adjacent cells, participating in physio and pathological processes [6]. The term “EVs” is used to denote lipid bilayer particles that are shed from virtually all organisms [7]. Currently, EVs comprise exosomes and microvesicles (MVs), which are differentiated by their biogenesis [8].

The biogenesis of exosomes involves the formation of multivesicular bodies (MVBs) containing intraluminal vesicles (ILVs) formed by budding from the endosomal membrane. ILVs can be degraded by the fusion of MVBs with lysosomes, or secreted into the extracellular space by the fusion of MVBs with the plasma membrane; in the latter event, they are called “exosomes,” having a diameter that varies from 30 to 100 nm. On the other hand, MVs are released directly into the extracellular medium through the outer budding of the plasma membrane, and are heterogeneous in size, ranging from 100 nm to 1 μm [6,9]. Due to the overlapping physical characteristics (such as size) between MVs and exosomes, it becomes challenging to separate these subpopulations. In the absence of specific markers of subcellular origin that are reliable in a given biological model, the ISEV recommends the use of the generic term “extracellular vesicle” (EV), or else that researchers name them based on technical-operational terms, such as use of “Large Extracellular Vesicles” (LEVs, which correspond mostly to MVs) and “Small Extracellular Vesicles” (SEVs, mostly exosomes). EVs act in cell communication by delivering their loads to recipient cells resulting in phenotypic changes that can affect the physiological state of these cells [6]. Due to their different origins, large and small vesicles could have different functions, and the methods to obtain them vary in efficiency [10]. 

The production of EVs by *G. intestinalis* and the role of Giardia EVs in the parasite-host interaction has been described. Kim et al. (2022) [11] showed that Giardia EVs improve clinical signs and reduce colon shortening in dextran sulfate (DSS)-induced colitis. The authors also observed that treatment with Giardia EVs suppressed neutrophil infiltration into colonic tissues, reducing inflammation. Our group showed that EVs produced by *G. intestinalis* are able to increase the adhesion of trophozoites to the surface of Caco-2 cells [12]. In another work, we described two populations of EVs produced by *G. Intestinalis*: the LEVs, mostly microvesicles, and the SEVs, consisting mainly of exosomes [13]. We also report that the inhibition of peptidyl arginine deiminase activity results in a decrease in the production of EVs by *G. intestinalis*, altering its ability to adhere to the host cell, and we show that only LEVs were able to restore the ability of the parasite to adhere to the host cell after treatment with the inhibitor.

The separation and purification of extracellular vesicles is not easy to accomplish, and this has been indicated by differences in protocols with respect to differential centrifugation utilized by different groups [14,15,16,17,18,19]. Sometimes, there may be non-representative populations of large or small vesicles in the samples being studied that do not allow us to understand the phenomena under analysis.

This work aimed to define a methodology based on differential centrifugation to enrich LEVs in order to better understand their role in the interaction of *G. intestinalis* with a host. In this study, we compared the different times of differential centrifugation required to achieve the enrichment of LEVs, using the parasite *G. intestinalis* as a study model.

## 2. Materials and Methods

### 2.1. Parasite and Cell Culture

The trophozoite forms of *G. intestinalis* (WB strain) were cultured in 13 mL of modified TYI-S-33 medium [20] (17.11 mM of NaCl; 7.12 mM of K_2_HPO_4_; 4.41 mM of KH_2_PO_4_; 3% yeast extract; 0.05% bovine bile; 55.55 mM of Glucose; 16.5 mM of L-Cysteine; 1.14 mM of Ascorbic acid) supplemented with 10% bovine adult serum (BAS) and 1% antibiotic (streptomycin), in a 15 mL Falcon tube at 37 °C.

Caco-2 human intestinal epithelium cells were cultured in RPMI 1640 medium, and supplemented with 10% fetal bovine serum (FBS) and 1% antibiotic (streptomycin) in culture flasks at 37 °C inside an incubator with 5% CO_2_, as previously described by our group [13]. The cells were passed every three days using trypsin until 80–90% confluency was attained.

### 2.2. Isolation of EVs

*G. intestinalis* cultured in the logarithmic phase was placed on ice for 15 min to release it from the tube. To induce the release of EVs, 1 × 10^6^/mL of the parasite was incubated in the TYI-S-33 medium without serum, to which1 mM of CaCl_2_ was added, at 37 °C for 1 h, in an incubator with 5% CO2. After the incubation time had elapsed, the tube supernatant was centrifuged at 425× *g* for 5 min to remove the cells. The supernatant from this first centrifugation was transferred to a new tube and centrifuged at 4000× *g* for 30 min to remove the cellular debris. Then, the supernatant was separated and used in different experiments; one part was centrifuged at 15,000× *g* for 1 h (15 K 1 h pellet), another part was centrifuged at 15,000× *g* for 4 h (15 K 4 h pellet) and the other part was ultracentrifuged at 100,000× *g* for 1 h and 30 min (100 K pellet). The pellet (containing the EVs) was resuspended in 1× PBS and stored at 4 °C for later analysis. 

Caco-2 human intestinal epithelium cells (2 × 10^6^/mL) were seeded in culture flasks and incubated for 24 h. After the incubation period, the cells were washed with RPMI medium without FBS, and then incubated in RPMI medium without FBS, plus 1 mM of CaCl_2_, at 37 °C for 1 h, in a 5% CO_2_ incubator. The EVs were isolated from the culture supernatant using the protocol described above.

### 2.3. Isolation of EVs at Acidic pH

The TYI-S-33 medium without serum was adjusted to pH 7 and pH 5, and *G. intestinalis* at a concentration of 1 × 10^6^/mL was incubated in this medium, with the addition of 1 mM of CaCl_2_, at 37 °C for 1 h. After the incubation period, the EVs were isolated following the protocol described above, with the exception of centrifugation at 100,000× *g* for 1 h and 30 min.

### 2.4. Characterization of EVs

The EVs were quantified based on their protein concentrations using the PierceBCA assay, and by nanoparticle tracking analysis (NTA-LM10 Nanosight, Malvern, UK), version NTA 3.4 Build 3.4.4, which reports the concentration and size distribution of the particles. For NTA, each sample was diluted 1:30 in PBS (1×) and subjected to a Nanosight, with readings taken in triplicate during 60 s videos with Detect Threshold 3. For analysis purposes, we considered large extracellular vesicles (LEVs) to be particles larger than 100 nm.

### 2.5. Transmission Electron Microscopy

*Giardia intestinalis* were centrifuged to generate a 15 K 1 h pellet and a 15 K 4 h pellet, and were then sonicated for 2 min in order to obtain a better aspect on the vesicles. Then, they were deposited on the grid for 10 min to achieve the adhesion of the sample. After this period, it was necessary to remove the excess of this sample, passing the base of the grid on a filter paper. Then, the process of fixation and negative contrast began. First, the grids containing the samples were fixed with 2.5% glutaraldehyde for 10 min and washed with 0.1 M sodium cacodylate 3 times through the deposition of the grid on a drop of this solution. It was necessary to dry the grid before proceeding to each subsequent wash. Finally, they were placed in 5% uranyl acetate for 3 min for the purpose of negative-contrast staining, washed quickly in Milli-Q water with a resistivity of 18.2 Ω, and dried. The properly prepared samples were submitted to analysis under the transmission electron microscope.

### 2.6. Uptake Assay

For the uptake assay, *G. intestinalis* EVs were isolated following the protocol described above, with the exception of centrifugation at 100,000× *g* for 1 h and 30 min. Also, the supernatant from the 15,000× *g* 1 h and the 15,000× *g* 4 h centrifugations were ultracentrifuged at 100,000× *g* for 1.5 h to generate a pellet containing small extracellular vesicles (SEVs 1 and SEVs 2, respectively).

For the flow cytometric analysis, Caco-2 cells were seeded at a density of 100,000 cells/well in 24-well plates for 24 h. *G. intestinalis* EVs (LEVs or SEVs) were incubated with 2 µL of DiI lipophilic membrane dye (1,1′-dioctadecyl-3,3,3′,3′-tetramethylindocarbocyanine perchlorate) in 1× PBS for 20 min in a dark at room temperature. After incubation, the dye was removed, 500 μL of bovine serum albumin (BSA) was added, and then it was centrifuged at 15,000× *g* for 1 h at 10 °C to wash the EV pellet. To demonstrate that the labeling of EVs was specific and not just for soluble DiI, the same amount of DiI was added to PBS, and the same centrifugations and treatments with EVs were maintained. Then, the cells were incubated with LEVs or SEVs (7 μg protein/mL) DiI-labeled for 6 h. Subsequently, the cells were washed 3 times with PBS, trypsinized and fixated in 0.2% paraformaldehyde. The samples were subjected to flow cytometric analysis on a FACSCanto (BD Biosciences). The data were analyzed with FlowJo.

For the confocal microscopy analysis, Caco-2 cells (100,000 cells/well) were incubated on sterile coverslips with LEVs or SEVs (7 μg protein/mL) that were DiI-labeled for 6 h. Caco-2 cell monolayers were also labeled for nuclei (DAPI, blue—ThermoFisher™, Waltham, MA, USA). After incubation, the cells were washed three times in cold PBS (1×), and fixed with 4% paraformaldehyde. The coverslips were washed with PBS (1×) and mounted with 10 μL of a Permount solution. Internalized EVs were detected using confocal microscopy (Nikon A1R HD Multiphoton Confocal, Nikon, Tokyo, Japan). The images were processed using FIJI Image J software 1.56. 

### 2.7. Statistical Analysis

To analyze the data, we used one-way ANOVA with Tukey’s post-test. The results were considered significant when *p* < 0.05. For statistical analysis, the GraphPad Prism Inc. version 6.1 program was used.

## 3. Results

### 3.1. A Modified Differential Centrifugation Protocol Produced an Enrichment of G. intestinalis and Caco-2 LEVs

**Figure 1:**
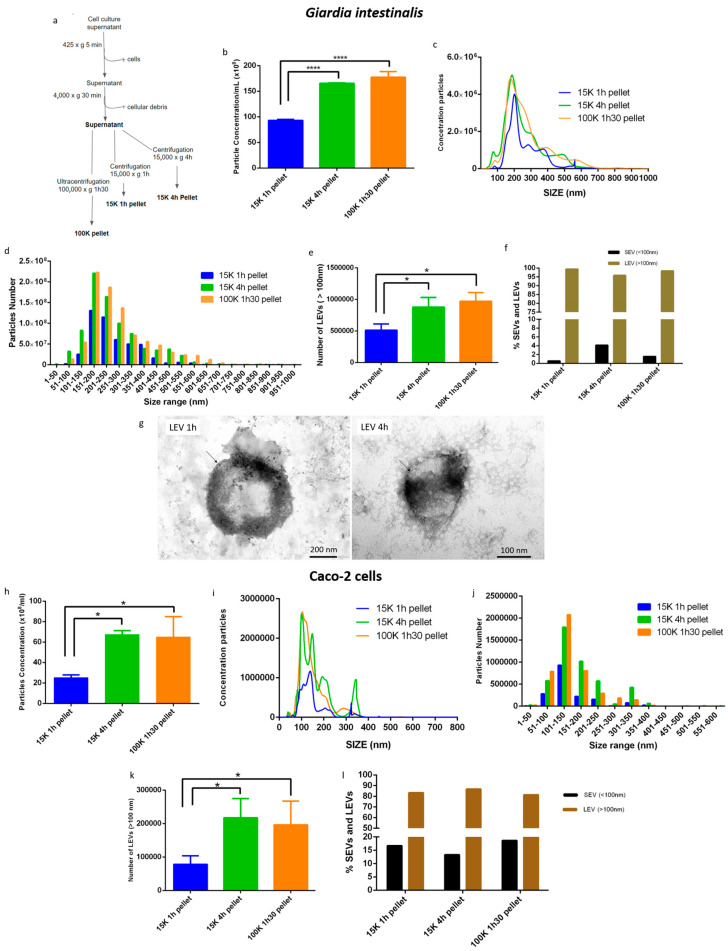
Enrichment and characterization of large extracellular vesicles (LEVs) released by *G. intestinalis* (**b**–**f**) and Caco-2 cells (**g**–**k**). (**a**) The protocol for enrichment of LEVs based on differential centrifugation. (**b**,**c**) Quantification of *G. intestinalis* vesicle concentration by nanoparticle tracking analysis (NTA). (**d**) Size distribution of *G. intestinalis* EVs. (**e**) Number of LEVs released by *G. intestinalis*. (**f**) Percentage of *G. intestinalis* small extracellular vesicles (SEVs) and LEVs. (**g**) Transmission electron microscopy of LEVs 1 h and 4 h; arrows point to regions where it is possible to observe the lipid bilayer of LEVs. (**h**,**i**) Quantification of Caco-2 cell vesicle concentration by NTA. (**j**) Size distribution of Caco-2 cell EVs. (**k**) Number of LEVs released by Caco-2 cells. (**l**) Percentage of SEVs and LEVs from Caco-2 cells. Asterisks indicate statistical difference between groups. **** *p* < 0.0001; * *p* < 0.01.

Using the protocol described in the materials and methods section, EVs of *G. intestinalis* were isolated by differential centrifugation at 15,000× *g* for 1 h (15 K 1 h pellet) or 4 h (15 K 4 h pellet), and by differential ultracentrifugation at 100,000× *g* for 1 h 30 (100 K 1 h 30 pellet) in order to separate EVs subpopulations (Figure 1a). The isolated EVs were characterized using nanoparticle tracking analysis (NTA). The NTA showed that the 15,000× *g* 4 h centrifugation was able to concentrate at the same levels of particle concentration as a direct ultracentrifugation (100,000× *g*, 1 h 30) (Figure 1b). The 15,000× *g* centrifugation for 1 h resulted in half the particles encountered in the 100 K pellet (Figure 1b), although the distribution profile remained similar, with a prominent peak of 200 nm EVs and a smaller peak corresponding to small extracellular vesicles (SEVs—particles below 100 nm) (Figure 1c). The three centrifugations analyzed concentrated more particles in the range of 151 nm to 250 nm, with higher concentrations of particles in the 100 K pellet and 15 K 4 h pellet (Figure 1d). We analyzed the amount of >100 nm particles (which are considered LEVs and contain mainly microvesicles) in the pellets, and the results showed that the 100 K 1 h 30 pellet and the 15 K 4 h pellet enriched more LEVs compared to the 15 K 1 h pellet (Figure 1e). 

Our data showed a good enrichment of LEVs in the condition of 15 K 4 h centrifugation, without altering the characteristics of the total population, and with the presence of only less than 5% of SEVs (Figure 1f). Figure 1g shows a transmission electron microscopy indicating that the EVs are intact.

We also analyzed the enrichment of LEVs in the 100 K 1 h 30 pellet, the 15 K 1 h pellet and the 15 K 4 h pellet in the Caco-2 cell model (Figure 1h–l). The NTA result showed that the 100 K pellet and the 15 K 4 h pellet concentrate at least two times more particles than the 15 K 1 h pellet (Figure 1h), confirming the result observed in *G. intestinalis*. The highest particle concentration peaks correspond mostly to LEVs (Figure 1i). All the protocols compared concentrated the majority of the particles in the range of 101 nm to 150 nm (Figure 1j). We analyzed the quantity of LEVs enriched in the three centrifugations (Figure 1k), and the results indicated that the 100 K pellet and the 15 K 4 h pellet also enriched more LEVs compared to the 15 K 1 pellet h in the Caco-2 cell.

### 3.2. Giardia Intestinalis Has a Higher Release of LEVs at pH 5, and Are Well-Enriched Using the Modified Method

**Figure 2:**
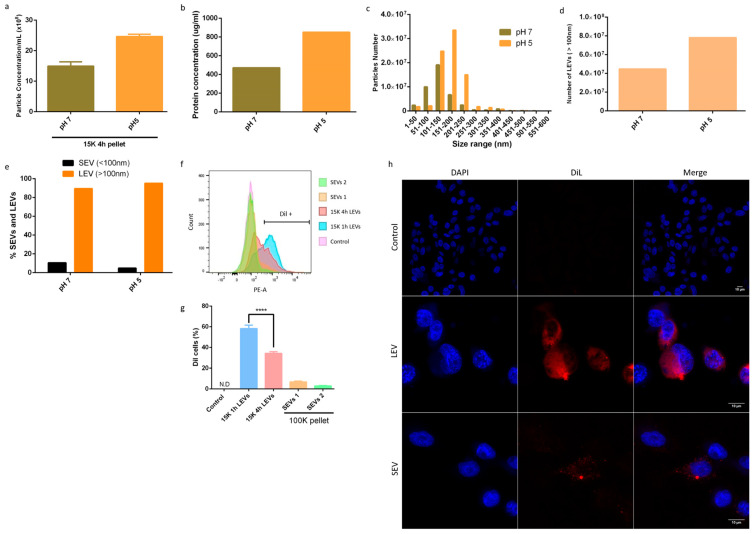
Enrichment of large extracellular vesicles (LEVs) by acidic pH (**a**–**f**) and uptake of EVs (**g**,**f**). (**a**) Quantification of *G. intestinalis* vesicle concentration by nanoparticle tracking analysis (NTA). (**b**) Micro BCA of *G. intestinalis* EVs. (**c**) Size distribution of *G. intestinalis* EVs. (**d**) Number of large extracellular vesicles (LEVs). (**e**) Percentage of *G. intestinalis* small extracellular vesicles (SEVs) and LEVs. (**f**) Uptake of *G. intestinalis* LEVs and SEVs by Caco-2 cells. (**g**) Percentage of Caco-2 cells positive for Dil staining of EVs. (**h**) Uptake assay of EVs labeled with Dil, visualized by confocal microscopy. Control represents Caco-2 cells that did not receive EVs (60× amplification). LEVs and SEVs indicate Caco-2 cells that received labeled EVs for 6 h (images represent 60× amplification). 15K 1h LEVs: LEVs isolated by centrifugation at 15,000× *g* for 1 h; 15 K 4 h LEVs: LEVs isolated by centrifugation at 15,000× *g* for 4 h; SEVs 4: SEVs isolated from the 15 K 4 h centrifugation supernatant; SEVs 1: SEVs isolated from the 1 h 15 K centrifugation supernatant. Asterisks indicate statistical difference between groups. **** *p* < 0.0001.

The secretion of EVs can be increased as a result of several conditions [21]. To evaluate the efficiency of the method, we decided to analyze the enrichment of LEVs under an acidic condition at pH 5, compared to the secretion of EVs at a normal physiological pH (pH 7) (Figure 2a–e). There was an approximate 30–50% increase in the number of particles and in the dosage of proteins obtained from EVs derived from the exposure of the parasite to pH 5, and isolated by centrifugation at 15,000× *g* for 4 h (Figure 2a,b). At pH 7, most of the particles ranged from 50 nm to 150 nm, while at pH 5, the particles were interestingly concentrated in the range of 150–250 nm (Figure 2c). The LEVs were at least two times more enriched at pH 5 than pH 7 (Figure 2d), while in both conditions the SEVs were produced similarly (Figure 2e).

We completed an uptake assay of *G. intestinalis* EVs by Caco-2 cells, comparing the internalization of the LEVs enriched by centrifugation at 15,000× *g* for 1 h (15 K 1 h LEVs) and those enriched for 4 h (15 K 4 h LEVs) and the internalization of isolated SEVs from the 1 h and 4 h 15 K centrifugation supernatant (SEVs 1 and SEVs 2, respectively) (Figure 2f,g). Flow cytometry results showed greater internalization of the 15 K 1 h LEVs compared to the 15 K 4 h LEVs. Both LEVs were more internalized than SEVs. We also analyzed the % of caco-2 cells that were EV +. We observed greater positivity in the cells that received the 15 K 1 h LEVs than cells that received the 15 K 4 h LEVs (Figure 2g), suggesting that the 15 K 1 h LEVs were more likely to facilitate the uptake. Moreover, the uptake was confirmed by confocal microscopy (Figure 2h). Confocal microscopy of Dil-labeled EVs by Caco-2 cells indicated that LEVs are more internalized than SEVs (Figure 2h).

## 4. Discussion

There are a variety of methods that have been employed to enrich EVs. Some are based on size (e.g., differential centrifugation, differential ultracentrifugation, ultrafiltration and size exclusion chromatography), density gradient (sucrose and iodixanol gradient), and immunoaffinity, among others [10]. Protocols for the enrichment of EVs differ greatly among research groups as a result of the notorious differences between large extracellular vesicles (LEVs), as can be seen in the following works: 14,000× *g* for 35 min [22]; 10,000× *g* for 30 min [23]; 100,000× *g* for 90 min [24] and 15,000× *g* for 1 h [13]. In this study, demonstrated a methodology based on differential centrifugation, which can be used to better enrich the LEVs, which can improve the understanding of the roles of these vesicles in the host–pathogen interaction. Our results suggest that LEVs produced by *G. intestinalis* can be better enriched by differential centrifugation at 15,000× *g* for 4 h. However, regarding purity, differential centrifugation at 15,000× *g* for 1 h contains less contaminating SEVs compared to differential centrifugation at 15,000× *g* for 4 h. With this protocol, LEVs can be enriched without the need for an ultracentrifuge, which is expensive equipment, and can be a limiting factor for many research groups that want to study extracellular vesicles.

This protocol may also help to further our understanding of factors involved with the secretion of the larger EV population, as we demonstrated with the acidic pH in *Giardia intestinalis.* The stimulation of EVS production by environmental factors has been studied in several models. Shao et al. (2018) [25] observed an increase in exosome production under hypoxic conditions in cancer cells. Moreover, it has also been described that glucose deprivation stimulates H9C2 cardiomyocytes to produce more EVs [26]. Low pH has been indicated as a factor that stimulates EVs, with high levels of cholesterol and caveolin-1 [27]. Evans-Osses et al. (2017) [12] observed that *G. intestinalis* is capable of producing microvesicles (mostly LEVs) under different environmental conditions, including at pH 5. Based on these findings, we stimulated the enrichment of LEVs by incubating *G. intestinalis* in a pH 5 medium. Our results suggest that at pH 5, *G. intestinalis* produces a greater quantity of large extracellular vesicles (LEVs-> 100 nm) compared to a normal pH. This result suggests an effect of acidic pH on the plasma membrane that results in the budding of larger EVs. Certainly, the mechanisms that control biogenesis and different specializations of vesicle subpopulations are still uncertain; however, a simple method for achieving the enrichment of LEVs opens the door to further investigations of the dynamics of vesicle release in different biological models.

Understanding the processes of biogenesis, release and uptake of EVs goes beyond basic science. Recently, EVs have gained attention in translational applications, from the discovery of biomarkers for diseases, to their roles in drug delivery systems. For example, the work by Gutierrez et al. (2022) [28] showed that EVs derived from the interaction of *T. cruzi* trypomastigotes with dendritic cells conferred protection to animals challenged with lethal infection by *T. cruzi*. Furthermore, the potential of EVs present in the serum of S. aureus osteomyelitis patients to facilitate the diagnosis has already been discussed [29]. Borgheti-Cardoso et al. (2020) [30] showed that EVs derived from red blood cells carrying antiparasitic drugs inhibited the in vitro growth of Plasmodium falciparum more efficiently than their free equivalents. The potential of EVs in translational applications is promising, but involves a long journey from characterization, standardization, scalability and clinical trials until their arrival to patients [31].

## Data Availability

The data presented in this study are available on request from the corresponding author.

## References

[1] Adam R.D. (2001). Biology of *Giardia lamblia*. Clin. Microbiol. Rev..

[2] Fink M.Y., Shapiro D., Singer S.M. (2020). *Giardia lamblia*: Laboratory Maintenance, Lifecycle Induction, and Infection of Murine Models. Curr. Protoc. Microbiol..

[3] Einarsson E., Ma’ayeh S., Svärd S.G. (2016). An up-date on *Giardia* and giardiasis. Curr. Opin. Microbiol..

[4] Cernikova L., Faso C., Hehl A.B. (2018). Five facts about *Giardia lamblia*. PLoS Pathog..

[5] Kulakova L., Galkin A., Chen C.Z., Southall N., Marugan J.J., Zheng W., Herzberg O. (2014). Discovery of Novel Antigiardiasis Drug Candidates. ASM J. Antimicrob. Agents Chemother..

[6] Van Niel G., D’Angelo G., Raposo G. (2018). Shedding light on the cell biology of extracellular vesicles. Nat. Rev. Mol. Cell Biol..

[7] Urabe F., Kosaka N., Ito K., Kimura T., Egawa S., Ochiya T. (2020). Extracellular vesicles as biomarkers and therapeutic targets for cancer. Am. J. Physiol. Physiol..

[8] Hill A.F. (2019). Extracellular Vesicles and Neurodegenerative Diseases. J. Neurosci..

[9] Kalra H., Drummen G.P.C., Mathivanan S. (2016). Focus on Extracellular Vesicles: Introducing the Next Small Big Thing. Int. J. Mol. Sci..

[10] Allelein S., Medina-Perez P., Lopes A.L.H., Rau S., Hause G., Kölsch A., Kuhlmeier D. (2021). Potential and challenges of specifically isolating extracellular vesicles from heterogeneous populations. Sci. Rep..

[11] Kim H.J., Lee Y.-J., Back S.-O., Cho S.-H., Lee H.-I., Lee M.-R. (2022). Treatment with Extracellular Vesicles from *Giardia lamblia* Alleviates Dextran Sulfate Sodium-Induced Colitis in C57BL/6 Mice. Korean J. Parasitol..

[12] Evans-Osses I., Mojoli A., Monguió-Tortajada M., Marcilla A., Aran V., Amorim M., Inal J., Borràs F.E., Ramirez M.I. (2017). Microvesicles released from *Giardia intestinalis* disturb host-pathogen response in vitro. Eur. J. Cell Biol..

[13] Gavinho B., Sabatke B., Feijoli V., Rossi I.V., da Silva J.M., Evans-Osses I., Palmisano G., Lange S., Ramirez M.I. (2020). Peptidylarginine Deiminase Inhibition Abolishes the Production of Large Extracellular Vesicles From *Giardia intestinalis*, Affecting Host-Pathogen Interactions by Hindering Adhesion to Host Cells. Front. Cell. Infect. Microbiol..

[14] Monguió-Tortajada M., Gálvez-Montón C., Bayes-Genis A., Roura S., Borràs F.E. (2019). Extracellular vesicle isolation methods: Rising impact of size-exclusion chromatography. Cell. Mol. Life Sci..

[15] Momen-Heravi F., Balaj L., Alian S., Trachtenberg A.J., Hochberg F.H., Skog J., Kuo W.P. (2012). Impact of Biofluid Viscosity on Size and Sedimentation Efficiency of the Isolated Microvesicles. Front. Physiol..

[16] Livshits M.A., Khomyakova E., Evtushenko E.G., Lazarev V.N., Kulemin N.A., Semina S.E., Generozov E.V., Govorun V.M. (2015). Isolation of exosomes by differential centrifugation: Theoretical analysis of a commonly used protocol. Sci. Rep..

[17] Lane R.E., Korbie D., Trau M., Hill M.M. (2017). Purification Protocols for Extracellular Vesicles. Methods in Molecular Biology.

[18] Chen A., He B., Jin H. (2022). Isolation of Extracellular Vesicles from *Arabidopsis*. Curr. Protoc..

[19] Stam J., Bartel S., Bischoff R., Wolters J.C. (2021). Isolation of extracellular vesicles with combined enrichment methods. J. Chromatogr. B Analyt Technol. Biomed. Life Sci..

[20] Keister D.B. (1983). Axenic culture of *Giardia lamblia* in TYI-S-33 medium supplemented with bile. Trans. R. Soc. Trop. Med. Hyg..

[21] Debbi L., Guo S., Safina D., Levenberg S. (2022). Boosting extracellular vesicle secretion. Biotechnol. Adv..

[22] Menck K., Klemm F., Gross J.C., Pukrop T., Wenzel D., Binder C. (2013). Induction and transport of Wnt 5a during macrophage-induced malignant invasion is mediated by two types of extracellular vesicles. Oncotarget.

[23] Muralidharan-Chari V., Clancy J., Plou C., Romao M., Chavrier P., Raposo G., D’Souza-Schorey C. (2009). ARF6-Regulated Shedding of Tumor Cell-Derived Plasma Membrane Microvesicles. Curr. Biol..

[24] Nievas Y.R., Coceres V.M., Midlej V., de Souza W., Benchimol M., Pereira-Neves A., Vashisht A.A., Wohlschlegel J.A., Johnson P.J., de Miguel N. (2017). Vesículas membranares do parasita Trichomonas vaginalis :caracterização e sua associ-ação com interação celular. Célula. Mol. Ciência Vida..

[25] Shao C., Yang F., Miao S., Liu W., Wang C., Shu Y., Shen H. (2018). Role of hypoxia-induced exosomes in tumor biology. Mol. Cancer.

[26] Garcia N.A., Ontoria-Oviedo I., González-King H., Diez-Juan A., Sepúlveda P. (2015). Glucose Starvation in Cardiomyocytes Enhances Exosome Secretion and Promotes Angiogenesis in Endothelial Cells. PLoS ONE.

[27] Hahm J., Kim J., Park J. (2021). Strategies to Enhance Extracellular Vesicle Production. Tissue Eng. Regen. Med..

[28] Gutierrez B.C., Ancarola M.E., Volpato-Rossi I., Marcilla A., Ramirez M.I., Rosenzvit M.C., Cucher M., Poncini C.V. (2022). Extracellular vesicles from Trypanosoma cruzi-dendritic cell interaction show modulatory properties and confer resistance to lethal infection as a cell-free based therapy strategy. Front. Cell. Infect. Microbiol..

[29] Deng S., Wang Y., Liu S., Chen T., Hu Y., Zhang G., Zhang X., Yu B. (2020). Extracellular Vesicles: A Potential Biomarker for Quick Identification of Infectious Osteomyelitis. Front. Cell. Infect. Microbiol..

[30] Borgheti-Cardoso L.N., Kooijmans S.A., Chamorro L.G., Biosca A., Lantero E., Ramírez M., Avalos-Padilla Y., Crespo I., Fernández I., Fernandez-Becerra C. (2020). Extracellular vesicles derived from Plasmodium-infected and non-infected red blood cells as targeted drug delivery vehicles. Int. J. Pharm..

[31] De Sousa K.P., Rossi I., Abdullahi M., Ramirez M.I., Stratton D., Inal J.M. (2023). Isolation and characterization of extracellular vesicles and future directions in diagnosis and therapy. Wiley Interdiscip. Rev. Nanomed. Nanobiotechnol..

